# Recognizing Posterior Accessory Muscles During Posterior Ankle Arthroscopy: A Case Report

**DOI:** 10.7759/cureus.101828

**Published:** 2026-01-19

**Authors:** Alexandre Castro, Raquel Cunha, João Teixeira, Pedro Atilano, Marta Gomes

**Affiliations:** 1 Orthopedics and Traumatology, Unidade Local de Saúde de Entre Douro e Vouga, Santa Maria da Feira, PRT; 2 Orthopedics and Traumatology, Hospital da Luz Arrábida, Vila Nova de Gaia, PRT

**Keywords:** accessory muscle tendon, chronic achilles tendon rupture, fibulocalcaneus internus, flexor hallucis longus, posterior ankle arthroscopy

## Abstract

The flexor hallucis longus (FHL) tendon is a key anatomical landmark in posterior ankle arthroscopy and a commonly used graft for chronic Achilles tendon rupture. Peroneocalcaneus internus (PCI) is a rare accessory muscle that is frequently overlooked due to its close anatomical relationship to FHL and its asymptomatic presence. Failure to recognize this variation may result in tendon misidentification, ineffective reconstruction, or neurovascular injury.

We report a 72-year-old woman with a chronic Achilles tendon rupture treated with endoscopic FHL transfer. Preoperative MRI demonstrated an accessory muscle running parallel to the FHL that was initially unrecognized. Intraoperatively, a tendon resembling the FHL was identified but showed no excursion with passive hallux motion, prompting further dissection. Distal tendon tracing confirmed the presence of a PCI inserting at the sustentaculum tali, allowing correct identification and safe harvesting of the true FHL. Retrospective MRI review highlighted distinguishing features of the PCI, including a separate muscle belly isointense to skeletal muscle, a posterolateral position relative to the FHL, and a calcaneal insertion.

This case emphasizes the importance of recognizing accessory muscles on preoperative MRI and applying strict intraoperative identification criteria. Awareness of the PCI and its distinguishing features can improve surgical planning, prevent reconstruction failure, and enhance safety during posterior ankle arthroscopy.

## Introduction

Flexor hallucis longus (FHL) has long been the landmark for posterior ankle arthroscopy [1]. Its identification is essential to avoid iatrogenic injury to the posteromedial neurovascular bundle [1]. Despite its usually uneventful identification, anatomic variations of muscles around the ankle may pose a difficulty and a risk for posterior ankle arthroscopy [2].

Supernumerary muscles are often overlooked during clinical evaluation; however, they can be clinically significant, as they may contribute to pain, compressive syndromes, and abnormalities in gait [3-6]. Several accessory muscles of the ankle have been described, but the ones that may cause confusion in identifying the FHL are the posteromedial accessory muscles deep to the deep aponeurosis of the leg [3-6]. There is a significant knowledge gap between imaging-based frequency data (~1%) and surgical recognition due to the asymptomatic nature, misidentification, and lack of reporting. This underscores the potential for under-reporting in clinical practice and literature [6].

Peroneocalcaneus internus (PCI) or fibulocalcaneus internus is a rare accessory muscle that originates from the lower third of the fibula, laterally to the FHL, running parallel to it and inserting at the base of the sustentaculum [4,7-9]. Distinguishing imaging characteristics on MRI include a muscle belly that is isointense to normal skeletal muscle, located posterolateral to the FHL and medial to the peroneal tendons. The muscle may displace the FHL medially and encroach on the neurovascular bundle within the tarsal tunnel. Its course and insertion differentiate it from the flexor digitorum accessorius longus, which typically runs more medially and inserts into the flexor digitorum longus or quadratus plantae [5,6]. In a case report, the muscle length was 14.12 cm with the muscle belly measuring 5.64 cm and the tendon 8.48 cm, but systematic MRI measurements (muscle belly length, tendon length, cross-sectional areas) have not been published [8].

The aim of this article is to raise awareness of accessory muscles during posterior ankle arthroscopy, as they may interfere with FHL identification. The presence of the PCI may displace the FHL, altering expected anatomy and increasing the risk of neurovascular complications. Preoperative MRI analysis, intraoperative structures identification, and imaging-surgical correlation are essential.

## Case presentation

An otherwise healthy 72-year-old woman presented to our consultation with a history of loss of impulsive force for the past six months. She recalled a history of acute calf pain and swelling that improved over time. Despite that, pain and a constant limp due to calf weakness didn’t resolve.

No clear palpable gap was present, although tenderness was reported. She presented with active plantar flexion of the foot but with loss of strength against resistance. Thompson’s and Matles test were positive. Gait assessment demonstrated an antalgic pattern with reduced push-off during the stance phase and difficulty performing a single-leg heel rise on the affected side, which the patient was unable to complete, in contrast to the contralateral limb.

Magnetic resonance imaging (MRI) revealed Achilles tendon rupture with a 5 cm gap. After failure of conservative treatment, the patient underwent endoscopic FHL transfer.

Operative technique

The patient is positioned prone with a sandbag under the contralateral thigh, and a thigh tourniquet is applied. Standard posterior arthroscopy as described by van Dijk et al. [1] was performed. After debridement, a longitudinally oriented tendon was found laterally to the FHL, but when passive dorsiflexion of the great toe was performed, motion wasn’t verified. For this reason, this muscle couldn’t be the FHL and was an accessory muscle. We kept performing our dissection until FHL was released.

**Figure 1:**
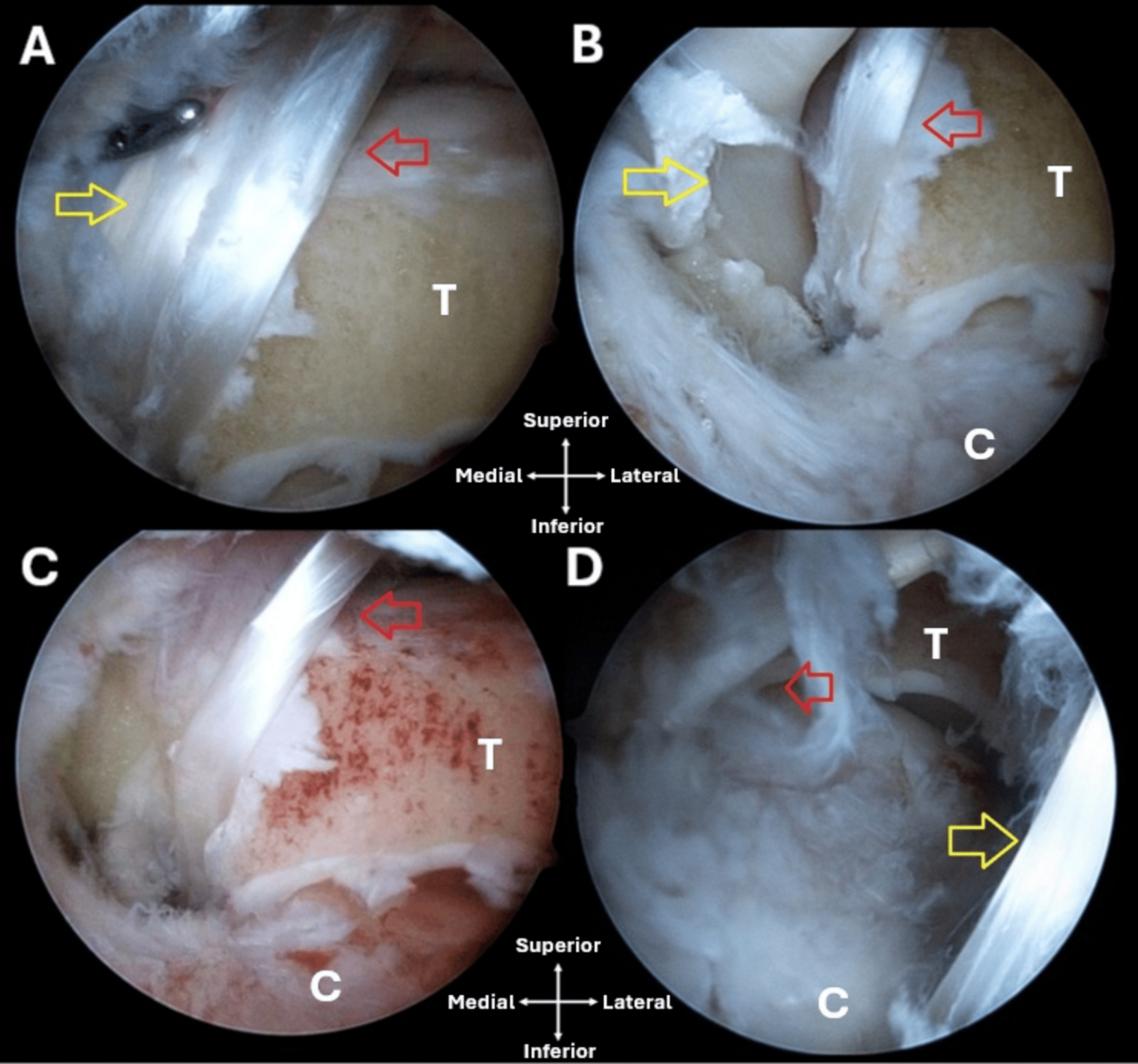
Posterior arthroscopy of the right ankle. (A) A longitudinally oriented tendon (red arrow) laterally to the FHL (yellow arrow). (B) FHL (yellow arrow) medially to the accessory tendon (red arrow). (C) The FHL was released, and the accessory tendon (red arrow) was left in place. (D) The FHL (yellow arrow) was transferred and fixed to the calcaneus, and the accessory tendon (red arrow) was left in place. FHL: flexor hallucis longus; T: talus; C: calcaneus

FHL was then harvested, prepared, and fixed in the calcaneus with an interference screw (Figure 1).

Portals were closed, and a below-knee splint with the ankle in plantarflexion was placed.

**Figure 2:**
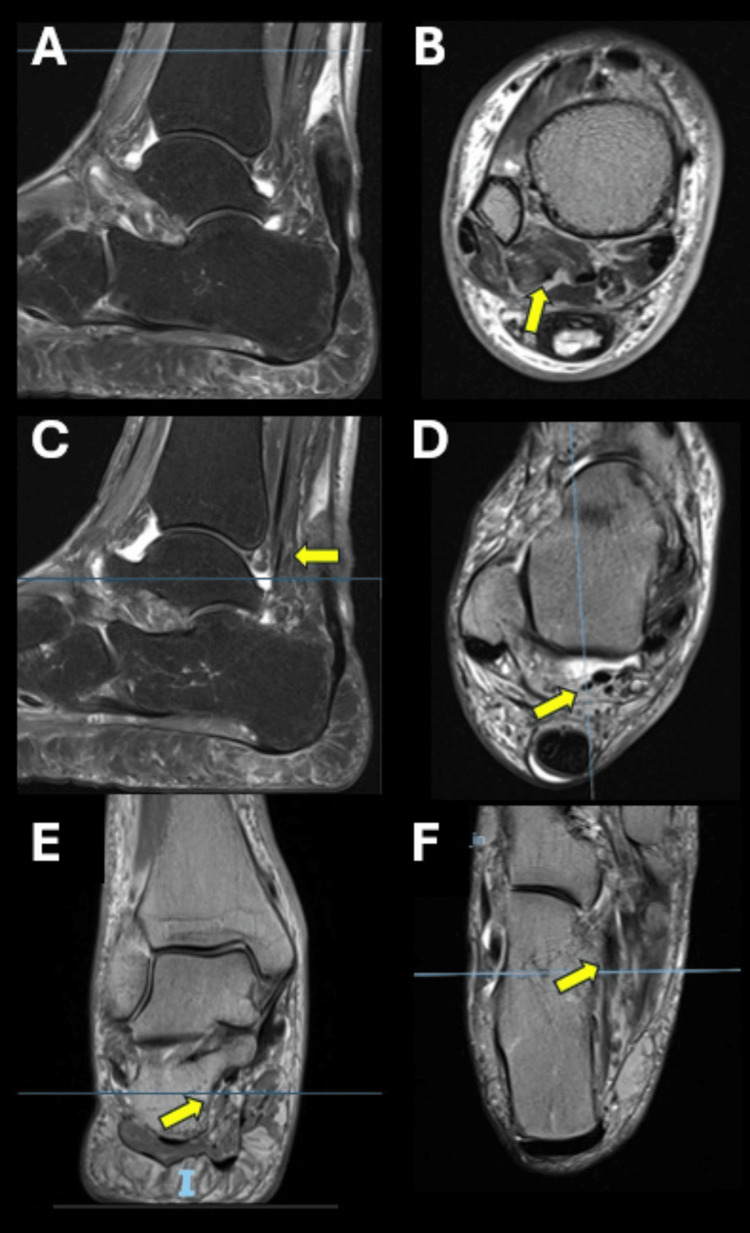
Right ankle MRI: (A-F) cross-sectional study including sagittal, coronal, and axial sequences demonstrating the peroneocalcaneus internus tendon (yellow arrows).

The MRI scan was retrospectively reviewed, and an extra posteromedial tendon was present running parallel to the FHL and inserting at the base of the sustentaculum. It was then identified as the PCI or fibulocalcaneus internus. Dedicated scrutiny of axial and coronal MRI planes, particularly at the level of the ankle joint, tarsal tunnel, and calcaneal insertion, may have suggested the presence of an accessory tendon distinct from the FHL (Figure 2).

The patient was discharged the following day and was submitted to a rehabilitation protocol.

## Discussion

The presence of accessory muscles may cause confusion in posterior arthroscopy. Its preoperative presence should be recognized, and the diagnostic algorithm proposed by Cheung [4] may help distinguish and identify them.

FHL is recognized as the structure to identify when performing posterior arthroscopy due to its proximity to the posterior tibial artery and tibial nerve [10]. The presence of the PCI or fibulocalcaneus internus, when unrecognized, may cause injury to the neurovascular bundle. The muscle may displace the FHL medially and encroach on the neurovascular bundle within the tarsal tunnel. On MRI, a muscle located posterolateral to the FHL and medial to the peroneal tendons should alert us to the presence of an accessory muscle [5,6].

Despite its unknown prevalence, the PCI was shown to be 1% common in 100 asymptomatic patients in an MRI study [9]. Its presence has been referred to as a possible cause for posterior ankle pain, impingement, and tarsal tunnel syndrome [8,11,12].

In the surgical treatment of chronic Achilles tendon rupture, the FHL tendon is commonly selected for transfer because of its strength, excursion, and line of pull, which closely replicate the function of the Achilles tendon. If the PCI tendon is mistakenly identified as the FHL and transferred instead, the reconstruction is going to fail and will not restore the powerful push-off required during gait, leading to persistent weakness and poor functional outcomes. This highlights the critical importance of accurate intraoperative tendon identification, particularly in the presence of anatomical variants, to avoid ineffective reconstruction and potential surgical failure.

As per our knowledge, only one article reported arthroscopic images of this muscle. Despite Lambert et al., according to the MRI and images description, suggested that those were most likely examples of the flexor digitorum accessorius longus [8]. For this reason, this is the first article reporting arthroscopic images of this muscle.

## Conclusions

This case highlights the critical impact that unrecognized anatomical variants can have on both imaging interpretation and surgical decision-making in posterior ankle arthroscopy. The presence of a PCI muscle closely paralleling the FHL created a realistic risk of tendon misidentification, which could have led to an ineffective reconstruction. By correlating preoperative MRI findings with intraoperative anatomy and carefully tracing the tendon to its distal insertion, the true FHL was safely identified and harvested for transfer. Misidentification of the FHL tendon could also result in neurovascular complications. Increased recognition of these variations may improve MRI interpretation, refine arthroscopic landmarks, and ultimately enhance the safety and reliability of endoscopic procedures.
